# Larotrectinib Demonstrates CNS Efficacy in TRK Fusion-Positive Solid Tumors

**DOI:** 10.1200/PO.19.00009

**Published:** 2019-05-16

**Authors:** Ezra Y. Rosen, Alison M. Schram, Robert J. Young, Mark W. Schreyer, Jaclyn F. Hechtman, Catherine A. Shu, Nora C. Ku, David M. Hyman, Alexander Drilon

**Affiliations:** ^1^ Memorial Sloan Kettering Cancer Center, New York, NY; ^2^ Weill Cornell Medical College, New York, NY; ^3^ Columbia University, New York, NY; ^4^ Loxo Oncology, Stamford, CT

## INTRODUCTION

TRK fusions are highly actionable drivers of oncogenesis that occur in a wide variety of cancers,^1^ including non–small-cell lung cancers^2^ (NSCLCs) and breast carcinomas.^3^ Larotrectinib, a selective TRK inhibitor, has marked and durable efficacy in TRK fusion–positive tumors regardless of histology, with an objective response rate of 80%,^4^ and is now approved for this tumor-agnostic indication in the United States. Disease control in the CNS is crucial because cancers that harbor TRK fusions can present with primary or metastatic intracranial disease. We present two cases in which the efficacy of larotrectinib in the CNS is highlighted.

## CASE REPORT 1

**FIG 1.**
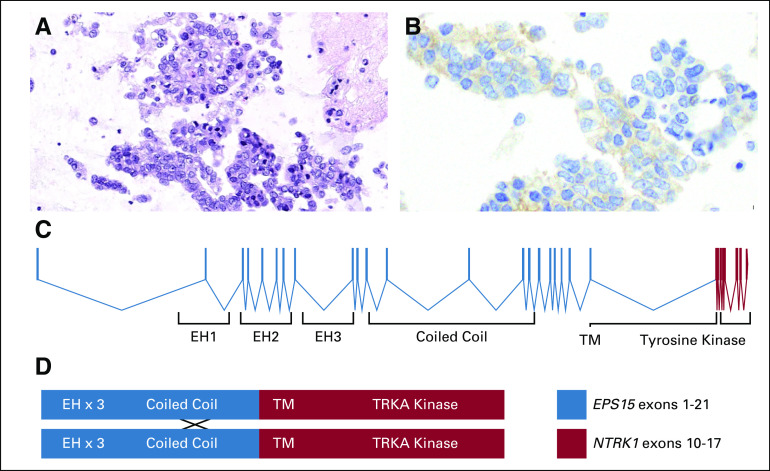
Pathologic and molecular features of a TRK fusion-positive lung cancer. Photomicrographs of (A) cytology from the patient’s lung adenocarcinoma and (B) positive immunohistochemistry results remarkable for cytoplasmic and membranous staining using a pan-TRK antibody are shown. (C) A map of the *EPS15-NTRK1* fusion protein that resulted from an in-frame fusion between *EPS15* exon 21 and *NTRK1* exon 10. (D) A schematic that shows the domains of the fusion protein depicted as possibly dimerizing at the coiled-coil region of *EPS15* where that protein is known to form homodimers.

A 76-year-old woman presented with a persistent cough. Extracranial imaging revealed innumerable bilateral pulmonary nodules; hilar and mediastinal adenopathy; and hepatic, adrenal, and osseous metastases. A magnetic resonance imaging scan of the brain, obtained in the absence of symptoms, identified more than 10 subcentimeter supratentorial-enhancing brain metastases. An endobronchial biopsy showed an adenocarcinoma consistent with a lung primary (thyroid transcription factor 1 positive, napsin A positive, and cytokeratin 7 positive by immunohistochemistry; Figs 1A and 1B).

Targeted exon sequencing of 468 genes with Memorial Sloan Kettering Integrated Mutation Profiling of Actionable Cancer Targets (MSK-IMPACT) was negative for known mitogenic drivers, including *KRAS*, *EGFR*, *BRAF*, *MET*, and *ERBB2* mutations and *ALK*, *ROS1*, and *RET* rearrangements. To further evaluate this apparent mitogenic driver-negative lung adenocarcinoma, targeted multiplex RNA sequencing (MSK Solid Fusion Panel) was performed. This identified a novel activating TRK fusion (in-frame fusion between *EPS15* exon 21 and *NTRK1* exon 10; Figs 1C and 1D).

**FIG 2.**
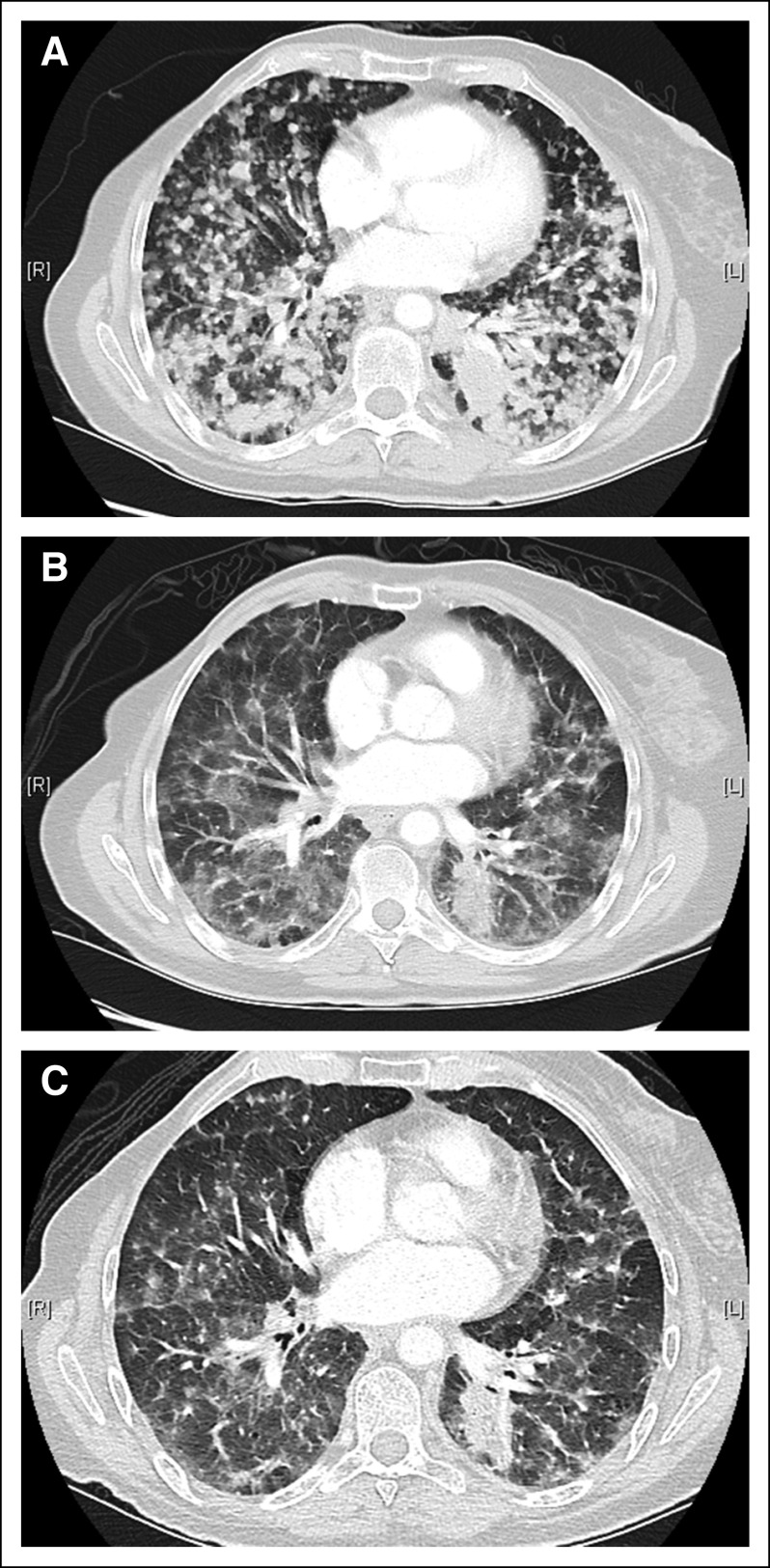
Extracranial response to larotrectinib in a patient with TRK fusion-positive lung cancer. (A) Baseline computed tomography imaging of the chest revealed innumerable bilateral pulmonary metastases that resulted in substantial dyspnea. Treatment with larotrectinib achieved a brisk clinical response within the first 2 weeks as evidenced by decreased shortness of breath. This was accompanied by a radiologic partial response to therapy that (B) was observed at 8 weeks and (C) was confirmed at 16 weeks. Previously solid and semisolid confluent nodules regressed substantially.

**FIG 3.**
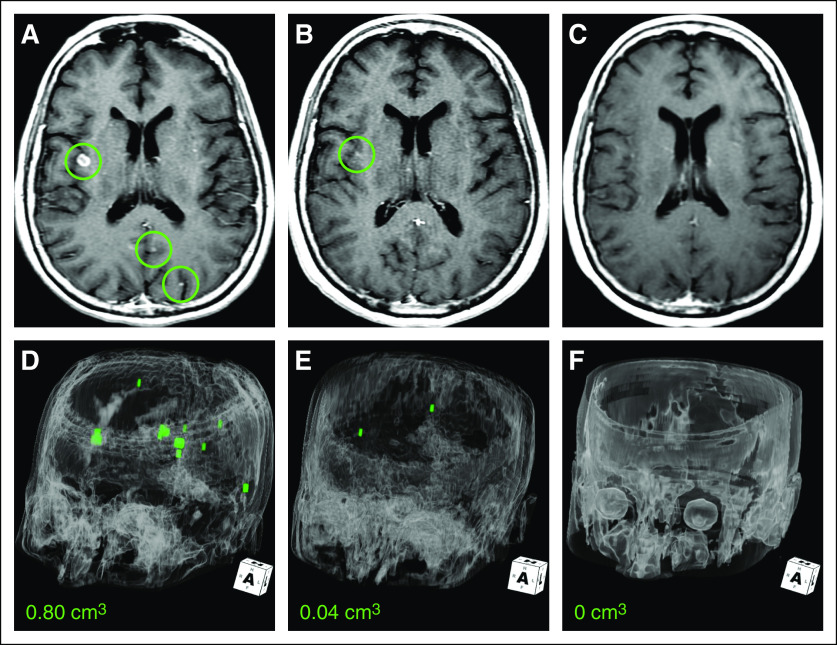
Intracranial response to larotrectinib in a patient with TRK fusion-positive lung cancer. Axial contrast-enhanced T1-weighted images at (A) baseline (brain metastases highlighted by circles), (B) 8 weeks, and (C) 16 weeks after treatment with larotrectinib was initiated demonstrate decreased and then resolved metastases in the right-side insula and left-side occipital lobe. Three-dimensional models at (D) baseline and (E) post-treatment at 8 weeks and (F) post-treatment at 16 weeks confirmed decreased burden of metastatic intracranial disease (segmented in green voxels and quantified at bottom). No residual disease was detectable at 16 weeks.

The patient consented to receive larotrectinib in a basket study for TRK fusion-positive cancers (NAVIGATE; ClinicalTrials.gov identifier: NCT02576431). No prior systemic or CNS radiation therapy was administered before trial enrollment. A brisk partial response to therapy was achieved at 4 weeks, which was subsequently confirmed at 8 weeks (–34% by Response Evaluation Criteria in Solid Tumors [RECIST] version 1.1; Fig 2), with regression of all areas of disease. This included near total resolution of all brain metastases at 8 weeks (95% reduction in aggregate tumor volume) and subsequently, a complete intracranial response by 16 weeks (Fig 3). She remains on larotrectinib with ongoing disease control and no major tolerability issues at more than 4 months.

## CASE REPORT 2

A 43-year-old woman presented with a palpable mass in her right-side breast. She underwent a right-sided therapeutic mastectomy and axillary lymph node dissection. Pathology showed a stage IIIC (T1cN3M0) multifocal invasive ductal carcinoma; the largest lesion measured 1.8 cm. Immunohistochemistry was positive for estrogen and progesterone receptors and negative for human epidermal growth factor receptor 2. All 13 lymph nodes examined were positive for disease. She was treated initially with adjuvant dose-dense doxorubicin, cyclophosphamide, plus paclitaxel and radiotherapy, after which she received anastrozole followed by bilateral salpingo-oophorectomy.

The patient developed recurrent, metastatic disease approximately 17 months after completing adjuvant radiotherapy. An abdominal lymph node biopsy confirmed recurrent, metastatic, triple-negative breast cancer that was also positive for androgen receptor expression. She received palbociclib and bicalutamide for 9 months, after which she briefly received capecitabine. Sequencing of an abdominal lymph node and a subsequently biopsied liver metastasis using MSK-IMPACT revealed the previously reported fusion between *LMNA* exons 1 and 20 and *NTRK1* exons 11 and 17, which was verified using targeted multiplex RNA sequencing.

**FIG 4.**
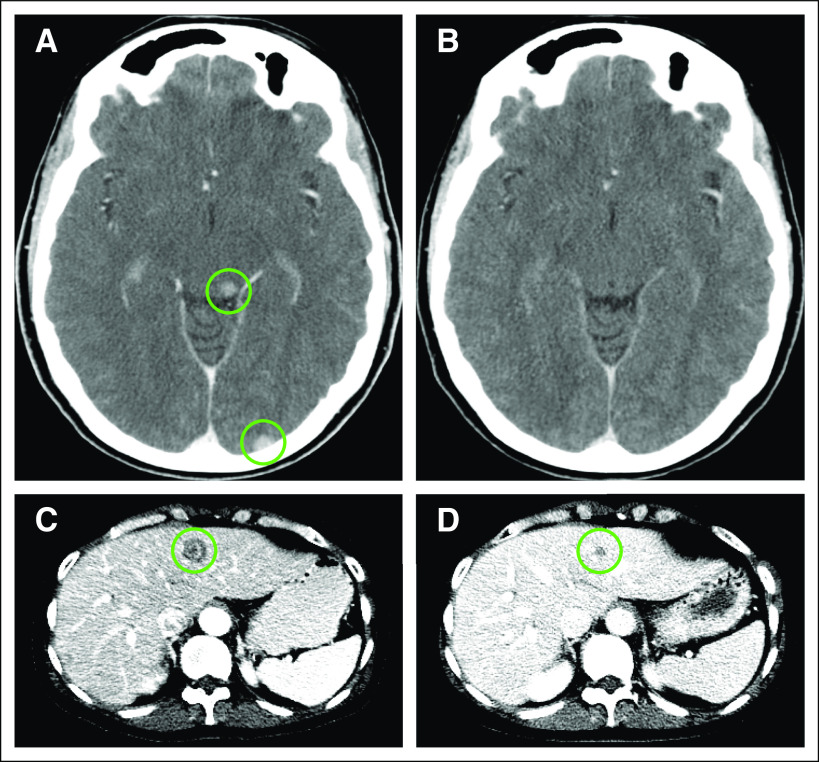
Response to larotrectinib in a patient with a TRK fusion-positive metastatic breast cancer. Baseline axial computed tomography imaging of the brain was performed with the addition of iodinated contrast that showed (A) metastatic brain disease (circles) and after 8 weeks of treatment with larotrectinib, (B) complete response. Radiologic partial response was also evident in the patient’s liver metastases at (C) 8 weeks, which continue at (D) 16 weeks. This partial response was visualized throughout her liver, adrenal, and bone metastases.

The patient consented to receive larotrectinib in the same basket study for TRK fusion-positive cancers (NAVIGATE). At enrollment, her disease was widely metastatic to liver, adrenal gland, lymph nodes, and bone. Brain imaging obtained in the absence of symptoms showed at least three subcentimeter brain metastases (Fig 4). A brisk and confirmed partial response by RECIST version 1.1 (−32%, −47%, and −56% at 4, 8, and 16 weeks, respectively) was achieved. Disease regression in the liver and adrenal gland and decreased uptake of hypermetabolic bony metastases were noted on serial imaging. Of note, her CNS metastases likewise regressed at the first follow-up assessment at 4 weeks, with a complete response in the CNS at 8 weeks (Fig 4). The patient remains on larotrectinib with good tolerability and disease control after 4 months of treatment.

## DISCUSSION

To our knowledge, this report is the first to showcase the intracranial efficacy of larotrectinib in metastatic TRK fusion-positive cancers of any histology. We report rapid and ultimately complete responses in previously untreated brain metastases with larotrectinib therapy in two patients with widely metastatic TRK fusion-positive lung and breast cancer. These cases demonstrate that larotrectinib can achieve clinically relevant CNS exposure, which highlights its role in the management of TRK fusion-positive cancers with intracranial disease.

Beyond NSCLCs and breast cancers, other TRK fusion-positive tumors have a proclivity for brain metastases.^1^ The intracranial activity featured here is consistent with prior work that did not identify CNS metastasis as a site of progression on larotrectinib across multiple tumor types^4^ and with a report that showed a response to larotrectinib in a TRK fusion-positive glioma.^5^ Additional clinical experience in patients with TRK fusion-positive cancers with CNS involvement ultimately will delineate the overall activity and durability of larotrectinib within the CNS.

Although this article represents what is to our knowledge the first report of intracranial responses to a selective TRK inhibitor in patients with TRK fusion-positive cancers and brain metastases, intracranial disease control also has been reported with multikinase TRK inhibitors. Entrectinib, a multikinase TRK, *ROS1*, and *ALK* inhibitor, displayed activity in a patient with a TRK fusion-positive lung cancer and brain metastases^6^ and in *ROS1* fusion-positive lung cancers with intracranial disease.^7^ Repotrectinib, a multikinase TRK, *ROS1*, and *ALK* inhibitor, achieved intracranial disease regression in untreated brain metastases in a patient with a *ROS1* fusion-positive NSCLC.^8^

Finally, this report represents the first description of the novel *EPS15-NTRK1* fusion. *EPS15* has been previously studied with regard to its role in secretion and endocytosis, and it is known to constitutively homodimerize at its coiled-coil region. This suggests that ligand-independent dimerization of the chimeric *EPS15*-TRKA oncoprotein may activate TRKA signaling.^9^
